# 3D Dynamic Spatiotemporal Atlas of the Vocal Tract during Consonant–Vowel Production from 2D Real Time MRI

**DOI:** 10.3390/jimaging8090227

**Published:** 2022-08-25

**Authors:** Ioannis K. Douros, Yu Xie, Chrysanthi Dourou, Karyna Isaieva, Pierre-André Vuissoz, Jacques Felblinger, Yves Laprie

**Affiliations:** 1 Université de Lorraine, CNRS, Inria LORIA, F-54000 Nancy, France; 2 Université de Lorraine, INSERM U1254 IADI, F-54000 Nancy, France; 3 Department of Neurology, Zhongnan Hospital of Wuhan University, Wuhan 430071, China; 4 School of ECE, National Technical University of Athens, 15773 Athens, Greece; 5 CIC-IT, Université de Lorraine INSERM 1433, CHRU de Nancy, F-54000 Nancy, France

**Keywords:** spatiotemporal atlas, generic speaker model, adaptive gaussian kernel

## Abstract

In this work, we address the problem of creating a 3D dynamic atlas of the vocal tract that captures the dynamics of the articulators in all three dimensions in order to create a global speaker model independent of speaker-specific characteristics. The core steps of the proposed method are the temporal alignment of the real-time MR images acquired in several sagittal planes and their combination with adaptive kernel regression. As a preprocessing step, a reference space was created to be used in order to remove anatomical information of the speakers and keep only the variability in speech production for the construction of the atlas. The adaptive kernel regression makes the choice of atlas time points independently of the time points of the frames that are used as an input for the construction. The evaluation of this atlas construction method was made by mapping two new speakers to the atlas and by checking how similar the resulting mapped images are. The use of the atlas helps in reducing subject variability. The results show that the use of the proposed atlas can capture the dynamic behavior of the articulators and is able to generalize the speech production process by creating a universal-speaker reference space.

## 1. Introduction

The differences in anatomy and articulatory strategy between speakers lead to very large variability of MRI images of the vocal tract, which prevents the creation of a unique 3D model that can represent any speaker. The creation of a generic approach and model that incorporates this variability starting from its construction is thus crucial. In the medical field, a popular approach to represent inter-subject image variability is the use of one or several atlases. In particular, this approach is very often used in brain studies for tasks such as automatic region segmentation, region labeling, etc. For instance, several atlases built from data of adults were used to automatically label and segment the brain regions of young prematurely born children [1]. Each of the adult atlases was registered to the target child image, and the final labeling and segmentation were based on a combination of the registration results. Such approaches facilitate the creation of automatically labeled atlases for young children by taking advantage of the availability of specific adult atlases and adapting them to the case of children for whom it is more difficult to acquire data.

There are several techniques to create an atlas or tackle the various issues that can appear during the creation process. One method to construct a brain atlas is to use affine registration to generate the anatomy-free reference space and then use non-rigid registration to create the “average brain” template [2]. Apart from creating a population-specific brain atlas, one can create a subject-specific brain atlas [3]. The main idea is that the similarity (in terms of image, gender, age, etc.) between the target subject and each subject of the rest of the population is computed, and this information is used as a weighting factor when creating the atlas of the target subject.

Another type of issue could appear during the use of the atlas, and more specifically, during the registration process of a new image to the atlas in order to extract atlas information for the specific subject. In order to map brain slices with severe histological artifacts to brain atlases, one can use an automatic method to identify the regions of artifacts and keep only the edge of the “correct” brain perimeter [4]. The estimated edge is then sampled, and these points are used as landmarks for point-to-point image registration with the atlas. The other possibility consists of mapping histological slices of the brain without brain reconstruction prior to registration since it can create artifacts [5]. The main problem that needs to be solved is how to find out the orientation used to acquire brain slices. In this approach, every histological slice is mapped to the atlas independently. The overall similarity is checked, and the atlas is rotated until the angle providing the maximal mapping similarity is found. This method is claimed to have similar or even better accuracy than previous algorithms for this task.

Even though these works are mainly focused on the static brain anatomy, there is also interest in the dynamics of the brain and how it evolves over time. For example, an anatomical dynamic brain atlas of the mouse was built by using brain scans of six mice at seven time points. The resulting dynamic atlas has the ability to provide a static atlas at those predefined time points [6]. The idea of predefined time points was further extended in [7], where a multidimensional atlas was presented that includes various contrast levels for every time point in addition to the baseline dynamic information at the predefined time points.

However, using predefined time points during atlas construction can be a limiting factor not only in the data acquisition process but also when studying brain evolution. In order to bypass this issue, a method was proposed in [8] which uses kernel regression to synthesize samples at any arbitrary time point by using all samples that are close to the target time point. Other methods were proposed, such as the one in [9], where first a dynamic model is built for each subject before combining all these models to create the final dynamic atlas space.

Apart from creating anatomical atlases, these methods can be used to create probabilistic atlases to estimate prior probabilities for automatic brain segmentation, such as in [10], where a 4-dimensional atlas was created based on affine transformations and gaussian kernels. Using kernels solves the problem of the dependency between data and atlas time points with the drawback that the resulting atlas time points could be synthesized from a variable number of data. This may result in differences in consistency and smoothness across the atlas time points. One solution is to improve the normal kernel method and use adaptive kernels instead, as proposed [11], which allows the same amount of data samples per synthesized atlas time point to be used.

Given the advancements and the flexibility in the atlas construction techniques, the atlas could be a powerful tool for investigating speech production. Earlier studies of speech articulators and especially the tongue used to be based on histological [12] or tagged cine-MRI of multiple subjects [13,14]. Later, however, some works exploited the atlas idea to create a motion field atlas of the tongue [15,16] for analyzing the correlation between the tongue muscles’ activities [17].

Dynamic atlases could provide valuable assistance in the study of speech production because, by construction, they involve the static (linked to the speaker anatomy) and dynamic (linked to the articulatory strategy) variabilities. The second aspect corresponds to rapid geometrical changes and, consequently, changes in the area function, which have a strong acoustic impact [18,19]. In the same conditions, atlas techniques could also improve speech imaging techniques [20] as it would allow low-quality images to be captured at a very high frame rate and the acquired image resolution to be increased by registering a high-resolution atlas to them. Indeed, spatio-temporal atlases are usually based on cine MRI to capture the 3D geometry of the vocal tract and its temporal evolution [21,22,23]. Such approaches rely on the repetition of a specific sentence to create the atlas. The underlying hypothesis is that the subject repeats the same sentence several times in exactly the same way, which requires prior training to speak by following a metronome. Additionally, the resulting atlas frame rate is fully dependent on the cine MRI acquisition frame rate.

In the present work, we proposed a method for constructing 3D dynamic atlases of the vocal tract using real-time MRI (rtMRI) of parallel sagittal planes at a high frame rate without requiring prior training. The main question addressed is whether it is possible to reduce speakers’ inter- and intra-variability by using the atlas space as a standard generic speaker. One of the contributions of our work is to employ the histological atlas creation approach [5] to collect the 3D information, using rtMRI [24] to acquire data, which offers a high frame rate and reduces the number of repetitions required by other techniques such as cine MRI. Such an approach is new for vocal tract atlases.

Another contribution is the use of the adaptive Gaussian kernel technique to create the atlas samples [11] with the advantage of making the atlas frame rate independent from the rtMRI frame rate. The proposed method thus gives more flexibility to control the resulting atlas parameters. Therefore, the same data can be used to create various atlases with different parameters without the need for new data acquisition every time. Finally, and this is a determining advantage in studying speech production, the atlas built with this method can be used as a reference speaker to reduce the variability between and within subjects.

Indeed, many works devoted to the production of speech from a general point of view are based on the implicit assumption that an articulatory model is built from a single speaker, which is often the case with the famous Maeda articulatory model [25], is valid for all speakers. This is a simplification that reduces the scope and validity of many studies. In our approach, on the contrary, we introduced the variability into the construction of the atlas itself, which therefore effectively covers a large speaker variability, provided that the speakers used are sufficiently diverse. Throughout the paper, the atlas thus refers to a specific model for a population of 3D (2D on parallel planes) vocal track dynamic images.

In this work, a dynamic vocal tract atlas was generated from rtMRI using the new proposed algorithm, and four-fold cross-validation with histogram matching was used to evaluate whether the atlas space is a valuable generic speaker model in order to reduce variability between speakers.

## 2. Method

Our method for constructing a dynamic atlas consisted of the following steps:

(1) **Acquire** 2D dynamic rtMRI parallel sagittal planes of the vocal tract during the production of several CVs;
(2) **Create** a subject-independent anatomical space based on a silent articulatory configuration;
(3) **Use this space** to remove the subject’s specific anatomical information from the dynamic images;
(4) **Combine** the previously created “anatomical neutral” dynamic images using piece-wise alignment and adaptive Gaussian kernel to create the dynamic atlas.

**Figure 1:**
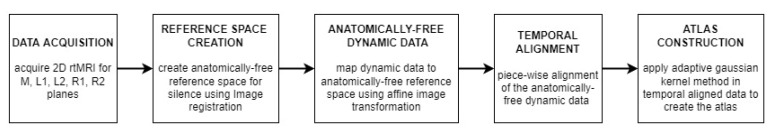
Overview of the atlas construction method.

The above steps are illustrated in the below diagram (Figure 1): 

### 2.1. Subjects

Subjects used in this study were four male and four female native speakers of French without any speaking or hearing problems. The average age was 27.25 years, with a standard deviation of 4.23 years.

### 2.2. Data Acquisition

The data were acquired on Siemens Prisma 3T scanner (Siemens, Erlangen, Germany) located in Nancy Central Regional University Hospital under the approved ethical protocol “METHODO” (ClinicalTrials.gov Identifier: NCT02887053). For the vocal tract measurements, 3D data were recorded using a multi-slice 2D T2 turbo spin echo (TR = 4610 ms, TE = 100 ms, flip angle = 15 degrees). The thickness of scan slices was 2 mm, and the pixel bandwidth was 445 Hz/pixel. Subjects were imaged with their mouth closed and breathing through the nose. For acquiring dynamic data, we used a 2D rtMRI sequence. Even though there are 3D dynamic sequences [26,27], 2D still offers better spatial and temporal resolutions. In our approach, we used radial RF-spoiled FLASH sequence [28,29] with TR = 2.22 ms, TE = 1.47 ms, FOV = 19.2 × 19.2 cm^2^, spatial resolution 1.41 × 1.41 mm^2^, flip angle = 5 degrees, and slice thickness is 8 mm. Pixel bandwidth is 1670 Hz/pixel. The number of radial spokes is 9, and the resulting image resolution is 136 × 136. The acquisition time was 44 sec. Images were recorded at a frame rate of 50 frames per second with the algorithm presented in [28] using a 64-channel head-neck antenna.

In order to capture 3D information with the 2D rtMRI sequence, we relied on the approach employed to construct brain histological atlases. Since the maximum width of the studied vocal tracts was 40 mm, we used 5 sagittal planes in total, the mid-sagittal one, two on the left and two on the right, with 0 mm frame spacing between them. For each subject, 5 contiguous sagittal planes (R2, R1, M, L1, L2) were acquired, covering the whole vocal tract. For each slice, the subject repeated the 12 CV syllables at a natural speed as instructed. In order to help the subject to reproduce the CVs in an identical way through the 5 repetitions, the text of the syllables was projected in the MRI for the duration of the acquisition. 

As described in [5], a major issue when dealing with slices is their orientation, which should be the same for all the speakers. Care was taken to ensure the exact sagittal alignment of the midsagittal slice for each subject to avoid misalignment problems previously reported in [5]. A way to solve this issue could have consisted of mapping the slices to an atlas and correcting them afterward. However, to the best of our knowledge, such an atlas does not exist. Therefore, instead of correcting slices, we tackled this issue one step before, during the real-time acquisition step, by using an MRI acquisition protocol designed to be as strict as we could make it to ensure that every time the target sagittal plane (i.e., R2, R1, M, L1, L2) was exactly the one being acquired.

**Figure 2:**
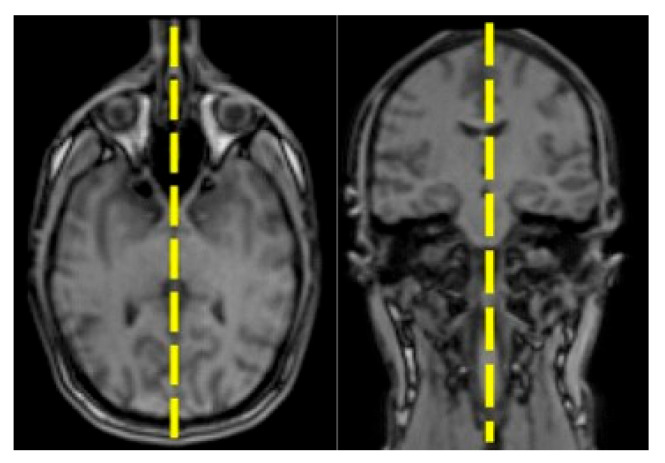
Definition of the midsagittal plane (M) using axial (**right**) and coronal (**left**) view.

**Figure 3:**
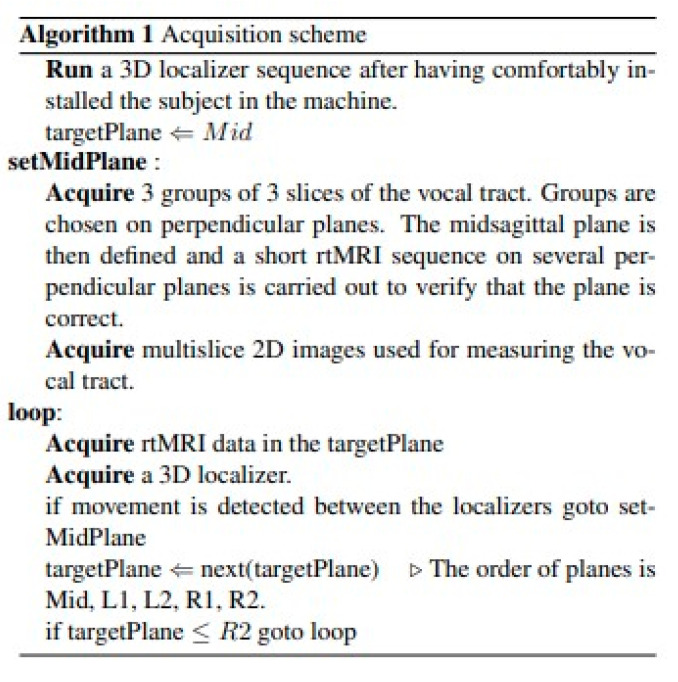
Algorithm for Acquisition.

The acquisition protocol was chosen to be as short as possible, keeping in mind that it should include a periodic check of the subject’s initial orientation and correct midsagittal positioning. The midsagittal plane was defined as the plane which passes in the middle of C2-C3 (in the coronal view) and separates the 2 brain hemispheres (in the axial plane). An overview of the midsagittal plane definition can be seen in Figure 2 and Figure 3, which show the overview of the acquisition algorithm.

This study focused on 12 CV syllables with C = {f, p, s, t} and V = {i, a, u}, i.e., /fi/, /fa/, /fu/, /pi/, /pa/, /pu/, /si/, /sa/, /su/, /ti/, /ta/, /tu/. The choice of these syllables was made so that we have two types of consonants, i.e., stops (/p/, /t/) and fricatives (/f/, /s/), two places of articulation, i.e., labials (/f/, /p/) and alveolars (/s/, /t/), in the context of the cardinal vowels (/i/, /a/, /u/). At this point, it is important to note that initially, we planned to include also the plosive /k/ in order to cover the three main places of articulation. However, probably due to the supine position in the MRI machine and the force of gravity, some subjects randomly pronounced either /k/ or /q/ during the acquisition even after proper instructions about the place of articulation. Given the difficulty of some subjects to accurately produce/k/through all the repetitions, we decided to exclude it.

In order to prevent coarticulation effects from previous random vocal tract positions, subjects were instructed to close the mouth and breath from the nose before articulating every CV so as to impose the same initial silence position every time. Additionally, the subject was instructed to finish every CV with /p/ so as to impose a minimal anticipatory coarticulation effect onto the vowel.

We chose /p/ because lips are the closest articulators to the head coil. The signal is thus stronger, and the image quality is very good for this articulator. Consequently, the contact between lips, which is used as a temporal landmark, can be detected with very good accuracy. Therefore, in practice, subjects uttered /sil//C//V//p/.

### 2.3. Vocal Tract Measurements

A practical way to increase the probability that subjects have different vocal tract sizes without directly measuring them is to measure their height before including them in our experimental protocol [30]. The smallest subject was 160 cm, while the tallest was 187 cm (average 174 cm).

**Figure 4:**
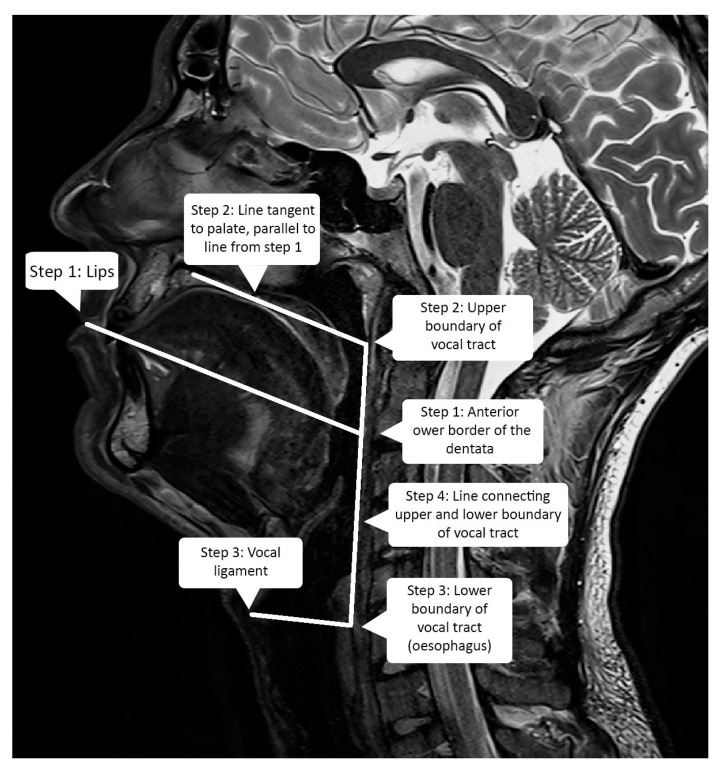
Vocal tract measurements algorithm.

**Figure 5:**
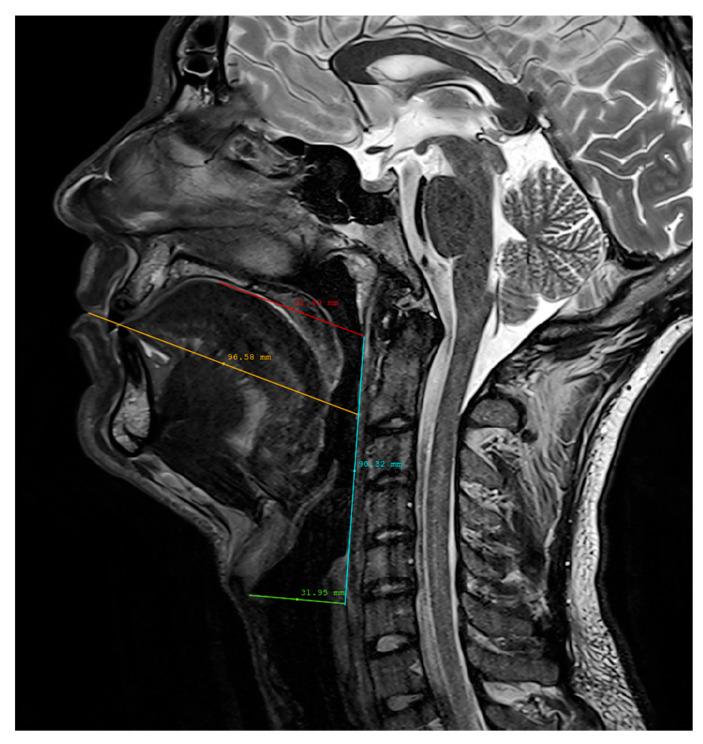
An example of the measurement algorithm applied to a subject.

In order to assess the ability of the atlas to be used as a standard generic speaker model, we measured the vocal tract dimensions of included subjects to ensure that there is enough variability in the dataset. Although several methods were proposed, for instance, using relative vocal tract/head position [31] or automatic articulatory landmark extraction [32], there is no standard method for measuring the vocal tract in terms of height, length, and depth since there is no strict definition of those measures due to the complexity of the vocal tract shape, which depends on the position, the articulated phoneme, etc. Therefore, we proposed the following method to measure the length and height of the vocal tract. It uses the midsagittal plane, and the first step is to draw a line from the outer touching point of the lips towards the anterior lower border of the body of the axis vertebra (Figure 4). The algorithm was manually applied to all speakers, and an example can be seen in Figure 5.

**Figure 6:**
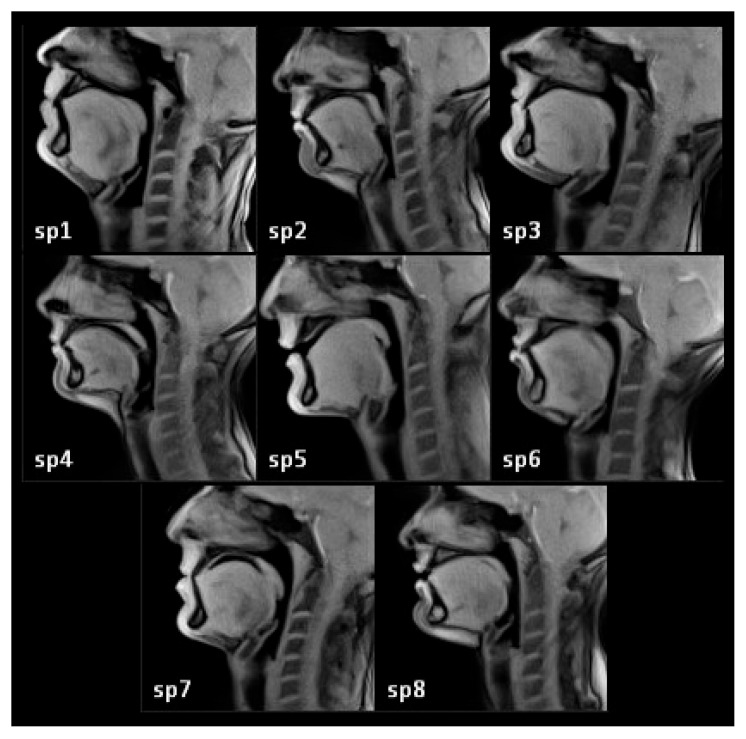
Midsagittal (M) frames for silence for all speakers (sp1–sp8 **left** to **right**, **top**–**down**). sp{odd} are male and sp{even} are female speakers.

The segment from the lips up to the intersection with the pharyngeal wall is defined as the length of the buccal cavity. The second step is to draw a line parallel to the previous one and tangent to the palate. The intersection point between this line and the pharyngeal wall is defined as the upper boundary of the vocal tract. The third step is to draw a line from the platform of the vocal folds until the esophagus. This point at the upper part of the esophagus is defined as the lower boundary of the vocal tract. The height of the vocal tract is defined as the distance between its lower and upper boundaries (Figure 4). In order to estimate the width of the vocal tract, all the sagittal planes were scanned, and the number of planes where the vocal tract is visible at the bottom of the pharyngeal cavity gives the width of the vocal tract. Table 1 shows the measurements for our group of subjects. The difference between the shortest and longest measure is 22 mm (σ = 6.5 mm) for the buccal cavity length and 25 mm (σ = 8.6 mm) for the height, i.e., more than 25% of these dimensions approximately. For the purpose of our task, we thus considered that these sizes exhibit sufficient variability [30]. Figure 6 shows the “silence” frames from all the speakers in the dataset.

### 2.4. Atlas Construction

The acquired dynamic films were manually labeled in order to achieve a better temporal segmentation. Image labeling was performed by a person with around 5 years of experience working with this type of image and was then checked by an expert with more than 15 years of experience in the field. For every /sil//C//V//p/ we only kept the /C/ and the /V/ part.

The stop onset is the first image where there is contact between the tongue tip and teeth for /t/, contact between lips for /p/, negligible lip movement for /f/ and negligible tongue tip movement for /s/. The vowel onset is the first image where the constriction is released, i.e., there is no more contact between the tongue tip ad teeth for /t/, and no more contact between lips for /p/, or the first image where there is increased lip movement for /f/ or the tongue tip for /s/. The vowel offset corresponds to the first image where lips are in contact because the subjects were instructed to articulate a /p/ after the second vowel. The average duration (number of frames at 50 Hz and in ms) per phoneme across all planes and speakers is given in Table 2.

The proposed construction algorithm relies on three hypotheses. First, all the slices are in the expected plane. For instance, all the central slices are in the mid-sagittal plane, and all the other sagittal slices are shifted from the mid-sagittal plane accordingly. This is a direct consequence of the very strict acquisition protocol we designed and the anatomical position we chose. As a consequence, images of one given plane and speaker can be compared and mapped with the corresponding images of all the other speakers. Anatomical differences between speakers could potentially affect this hypothesis all the more since a potential error can stack as one moves further from the midsagittal plane. However, we expect this error not to be significant because we moved just two slices away at most from the mid-sagittal plane and the slice thickness was big enough so that the outer parts of the vocal tract (in the sagittal direction) lie within the R2 and L2 planes for all subjects.

The second hypothesis is that the order of events is the same for all the speakers, which is expected and reasonable at the scale of an isolated CV.

Third, due to the frame rate of 50 Hz, small piece-wise linear extensions or compressions of the images in time do not significantly affect the dynamics of articulation.

For describing the construction of the atlas silence space, we refer to the midsagittal plane for simplicity unless it is specified differently. The process presented below for the midsagittal plane was repeated for all the other planes. Before every image transformation or averaging in this work, histogram matching was performed to transform the histogram of the moving image to one of the reference images. This is intended to compensate for intensity differences between images (Seghers, 2004).

The atlas construction process can be divided into four major steps:

(1) **Create** the anatomically free reference space;
(2) **Make** dynamic data anatomically free;
(3) **Align** data temporarily;
(4) **Synthesize** the atlas samples.

The objective of step 1 was to make the data anatomically neutral. By anatomically neutral, we mean that data are independent of anatomical variability and correspond to a virtual neutral speaker. For this purpose, we used a silence frame during breathing at a resting position before speakers start recording the CV (as described in the protocol, i.e., breathing from the nose with a closed mouth and without any visible articulatory movement) from all N speakers in order to create the reference anatomically free space. The average histogram was computed, and all the images’ intensities were transformed so that their histogram would match it [33]. For image registration, the transform used (*T*(*x*,*y*) with *x*,*y* being the image coordinates) is composed of two parts, the global and the local one.

(1) $$T\left(x,y\right)=T_{global}\left(x,y\right)+T_{local}\left(x,y\right)$$

In our case, an affine transformation was used for *T_global_* (*x*,*y*) and a cubic B-spline tensor product on control point grid transformation for *T_local_* (*x*,*y*) [34]. Therefore,

(2) $$T_{local}\left(x,y\right)=\sum_{l=0}^{3}\sum_{m=0}^{3}B_{l}\left(u\right)B_{m}\left(v\right)\phi_{i+l,j+m}$$

where $\phi_{i,j}$ are the control points with *δ_x_*_,_ *δ_y_* the spacing between them.

(3) $$i=\lfloor x/\delta_{x}\rfloor -1$$

(4) $$j=\lfloor y/\delta_{y}\rfloor -1$$

(5) $$u=x/\delta_{x}-\lfloor x/\delta_{x}\rfloor$$

(6) $$v=y/\delta_{y}-\lfloor y/\delta_{y}\rfloor$$

where *Bl*, *Bm* is the *lth* and *mth* B-spline base function [35]. Each image was registered to all other N−1 speakers’ images using the described non-rigid B-spline-based transformation using the image registration function of the MATLAB toolbox “B-spline Grid, Image and Point based Registration” [36].

This toolbox was used for all the transformations performed in this work. For every image, we obtained N−1 transforms. The average transformation (without any further weighting) was computed for every image, and this average transformation was applied to the corresponding image to produce the anatomical free version, which is image dependent. Finally, all the N image-dependent anatomical free spaces are truly averaged to create the final reference space (image independent, anatomically neutral).

**Figure 7:**
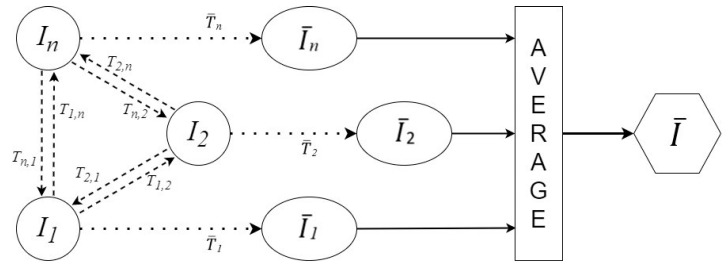
Creating the reference space. Every *ith* silence image is registered to all others; the computed transformations are averaged to give ${\bar{T}}_{i}$ and applied to the *ith* image to obtain ${\bar{I}}_{i}$. The resulting images are averaged to get the final reference space image $\bar{I}$.

More precisely, for the *i*th silence image from the set of silent images {I_1…n_}, the transformations $T_{i,j},i\neq j$ were computed and averaged to provide the average transformation ${\bar{T}}_{i}=\frac{1}{N-1}\sum_{j=1\ldots n,i\neq j}T_{i,j}$. Finally, the final reference space was created by applying the ${\bar{T}}_{i}$ transforms to the corresponding images and averaging them $\bar{I}=\frac{1}{n}\sum_{i=1\ldots n}{\bar{T}}_{i}\left(I_{i}\right)$ with ${\bar{T}}_{i}\left(I_{i}\right)≃{\bar{I}}_{i}$. A visual representation can be seen in Figure 7.

Step 2 intended to make the data anatomically free. First, the images’ histogram of all the CVs was matched with the histogram of the reference, and all the CV images were then transformed to the reference space using only an affine transformation (one for each image of all the CV images of all the speakers) computed with the same MATLAB function as in Step 1 because it transforms the anatomy of the data to the reference anatomy but keep the vocal tract position variability, i.e., the position of the articulators [10].

Step 3 intended to process the anatomical free data for applying the adaptive kernel technique. For each CV, all the planes of all the speakers were used to specify the corresponding average C and V duration. These values were set as the time reference durations for each of the C and V of the atlas. Data were then piece-wise linearly aligned to those CV time duration values using rtMRI frame rate to pass from the frame space to the time domain in order to compute the global time.

**Figure 8:**
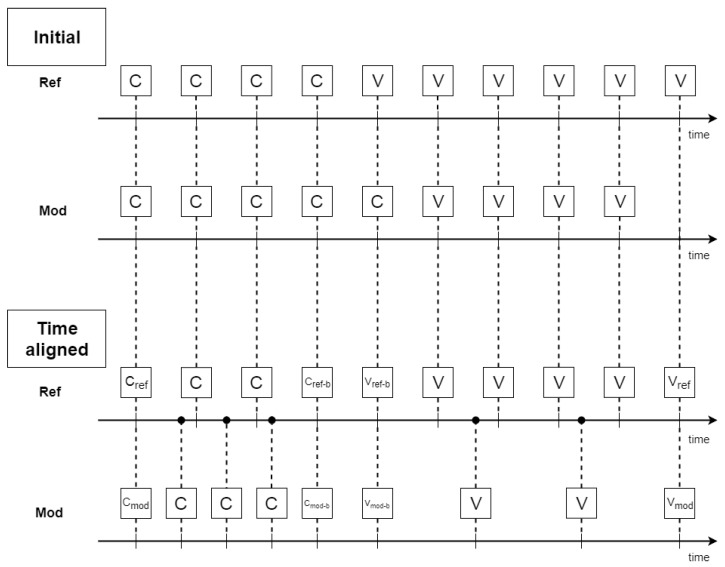
Piece-wise time alignment. Mod is the CV whose duration is to be modified in order to match the duration of the reference (Ref) CV. On the top are both CVs before time alignment (Initial), and on the bottom is the time-aligned version of the Mod CV with the Ref CV.

For example, in order to align a CV to be modified to a referenced CV, the C and V parts of the modified CV were independently and linearly extended or compressed until the duration of both C and V of the modified CV matched with those from the reference CV. This alignment technique (see Figure 8) is intended to achieve time alignment so as the duration of the modified (Mod) CV is that of the reference (Ref) CV, but not to map each frame of the reference CV to one of the current CV. In practice, this procedure creates one anatomical free image series for each of the 12 CVs from the image series of all speakers for the same CV by putting all frames in a global time scale based on the time stretching or compressing defined by the piece-wise linear alignment. It should be noted that the resulting series may have multiple frames at the one-time point and that samples are not homogeneously distributed across time.

Step 4 consists of synthesizing the atlas images from the global series of images, i.e., the 12 CVs involved in this work, by using the adaptive Gaussian kernel method [11]. The word “adaptive” refers to the width of the Gaussian kernel so that the same number of samples is used every time. The core idea was to generate the atlas image at a given target time point from *k* images in the global series located in the vicinity of the target time point. *k* is a pre-specified number of samples to choose the closest relevant samples, and the resulting image is the Gaussian weighted average of the *k* samples. This way, the resulting synthesized images are sharper and less blurry.

**Figure 9:**
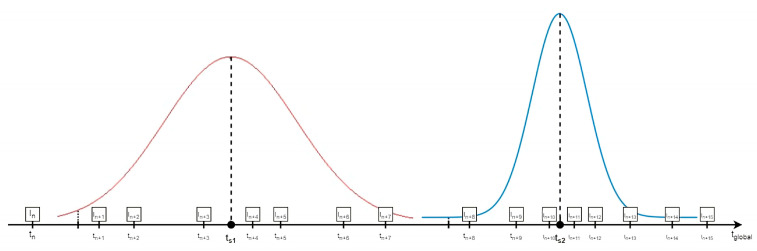
Adaptive Gaussian kernel technique. The width of the Gaussian is adapted based on the distance between the desired synthesis time points (ts1, ts2) with the available samples I_i_. The number of samples contributing to frame generation is stable.

The advantages are that the atlas frame rate is independent of the data acquisition frame rate and that the atlas sampling may not be regular since the time points can be chosen freely. Theoretically, the initial sampling rate has some influence, but the initial frame rate is high enough to study all common speech tasks [37]. However, the number of samples used to synthesize the images and the parameters of the Gaussian weights should be tuned. In [11], the number of samples was chosen as a function of the number of subjects available in the vicinity of the target time point and could vary substantially, i.e., from 3 to 25, because the number of subjects recorded depended on the time and the phenomenon monitored was much slower. Thus, when many subjects were available, the gaussian was sharp and conversely wider when fewer subjects were available. In our case, the number of subjects was constant, i.e., 6, and consequently, the number of samples available was almost constant if we consider that the dynamic variability is limited. We tested several choices and set k to 7 atlas samples within a window of 20 ms, which is the recording period and is expected to be sufficient for our study [37]. The Gaussian weighting was designed so that its mean value is the selected time point *τ* to be synthesized, and the standard deviation was tuned so that the weight of the farthest *k* sample *τ_f_* from the center is 0.35 of the maximum value of the Gaussian distribution. Therefore, the parameters of the Gaussian distribution are *µ = τ* and $\sigma =\sqrt{-{\left(\tau -\tau_{f}\right)}^{2}/\left(2*ln\left(0.35\right)\right)}$ [11]. Table 3 shows the parameter values used in the adaptive Gaussian kernel method. This approach is illustrated in Figure 9.

## 3. Validation

In order to evaluate the results, four-fold cross-validations were carried out using six subjects for training and two subjects for testing for every fold. In every fold, the two test subjects were chosen to be of a different gender to obtain results for both genders. Both of the test CVs are piece-wise linearly temporally aligned with the corresponding atlas CV. For each frame of each atlas CV, the temporally closest frame of the corresponding test CV is selected. It is thus possible for a test frame to be used more than once while some others may not be used at all. At this point, for each CV, each atlas frame is linked to two frames of the corresponding CV, i.e., one for the two test subjects. 

**Figure 10:**
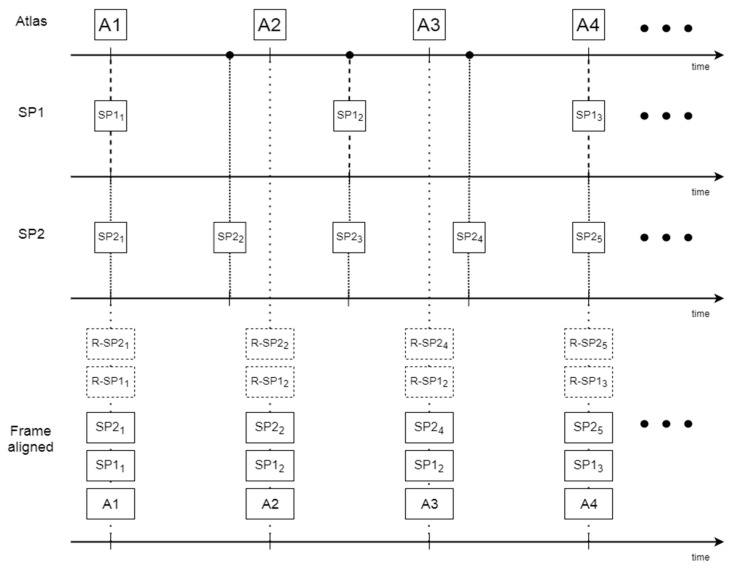
Frame alignment used for tests. A represents the atlas frames, and SPi_j_ original frames j for speaker i and R−SPi_j_ the registered framed within the atlas space.

**Figure 11:**
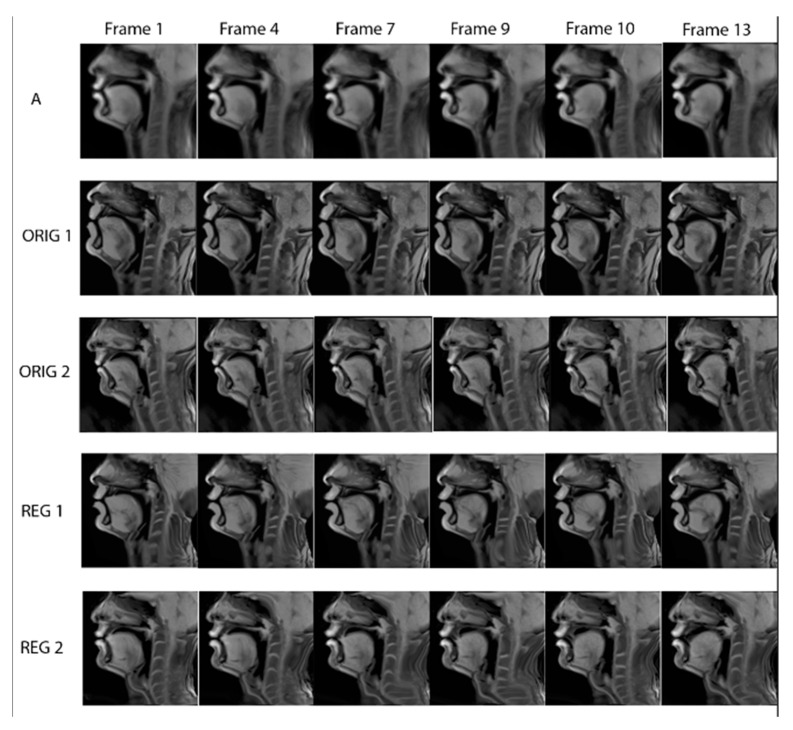
The midsagittal frames of the atlas for /tu/ with the corresponding test subject frames before and after transformation with the atlas.

All the frames linked with the same atlas frame form a stack of images, as seen in Figure 10. Each stack includes an atlas image and the corresponding images of **(i)** speaker 1 image without registration, **(ii)** speaker 2 image without registration, **(ii)** speaker 1 image after registration, **(iv)** speaker 2 image after registration. Examples of every stack of images in the midsagittal plane for /tu/ can be seen in Figure 11. Histogram matching was applied so that the histograms of the linked images with one atlas frame fit its histogram. Test images were mapped to the atlas image using the B-spline non-rigid transformation (the same technique as that used for construction). For example, images of row A from Figure 11 are the reference images of the atlas for /tu/. Images from rows ORIG 1 and ORIG 2 were mapped to those of row A, and the resulting images were shown in rows REG 1 and REG 2. The similarity between the original images (ORIG 1 and ORIG 2) and those of row A for all frames of all planes is computed. The similarity between the transformed images (REG 1 and REG 2 rows) was also calculated to check that the similarity increased after registration.

The idea of this procedure is to transform any given image of a target speaker’s CV as close as possible to the corresponding atlas image. We used cross-correlation as a similarity measurement between images mapped from the atlas and original images [11]. The cross-correlation value is normalized by the auto-correlation of the atlas frame. More precisely, for each stack of images, A is an atlas image, *O*_1_ and *O*_2_ are the original images of speaker 1 and speaker 2, and R1 and R2 are the corresponding registered images to the atlas. All images represent *M* × *W* matrices of pixel density values, with M and W being image dimensions. Before registration with the atlas (BA), the similarity (with zero-padding) is defined as:

$$BA=\frac{max\sum_{m=0}^{M-1}\sum_{w=0}^{W-1}O_{1}\left(m,w\right)O_{2}\left(m-k,w-l\right)}{max\sum_{m=0}^{M-1}\sum_{w=0}^{W-1}A\left(m,w\right)A\left(m-f,w-g\right)}$$

With

−(*M* − 1) ≤ *k*, *f* ≤ *M* – 1

−(*W* − 1) ≤ l, *g* ≤ *W* − 1

After registration, the similarity (with zero-padding) is defined as:

$$AA=\frac{max\sum_{m=0}^{M-1}\sum_{w=0}^{W-1}R_{1}\left(w,n\right)R_{2}\left(w-t,w-c\right)}{max\sum_{m=0}^{M-1}\sum_{w=0}^{W-1}A\left(m,w\right)A\left(m-f,w-g\right)}$$

With

−(*M* − 1) ≤ *t*, *f* ≤ *M* − 1

(*W* − 1) ≤ *c*, *g* ≤ *W* − 1

These measurements are averaged across space and time in order to produce Table 4. Columns 2 and 4 are the averages of BA and AA, respectively, and column 3 and 5 are the corresponding standard deviations.

## 4. Results

**Figure 12:**
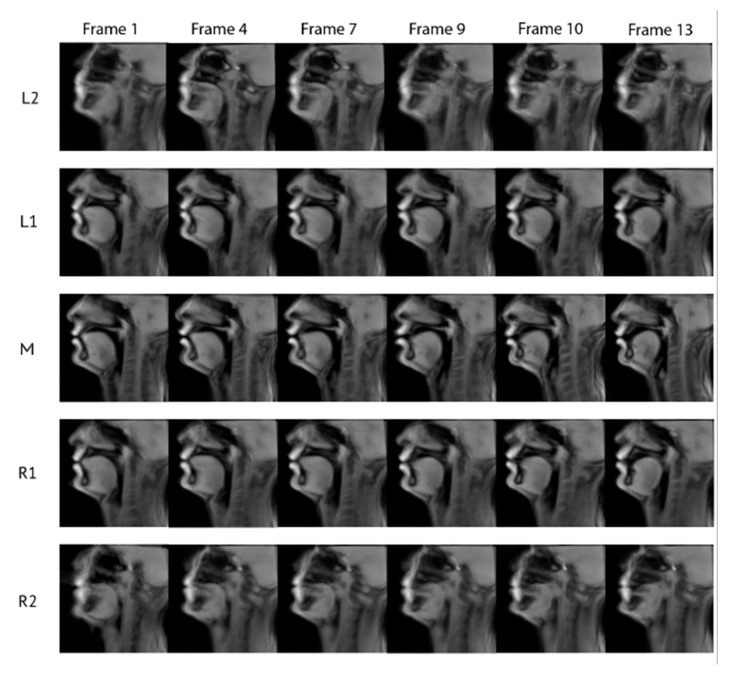
Frames 1, 4, 7, 9, 10, 13 of the atlas planes without sp5, sp6 for /tu/.

The methods presented above regarding the atlas construction were applied to the acquired data on all five planes. During the atlas construction process, small time variations appeared during the various registration processes due to the fact that, by nature, some speakers are anatomically more similar/different from each other. Figure 12 presents examples of frames from all sagittal planes in the atlas space for /tu/. The visual assessment confirms that the synthesized images represent the natural vocal tract position with the expected dynamics. This is further quantitively supported by the numerical results in Table 4. As it can be seen from Table 4, the average similarity between the images after applying the atlas is increased while the standard deviation decreases (col. 4, 5) compared to the similarity and the standard deviation without the atlas (col. 2, 3). Figure 10 shows the midsagittal frames of the atlas with the corresponding frames of the test subjects before and after atlas transformation. The places of articulation are clear for both /t/ and /u/.

We can see the dynamics of the tongue starting from the very beginning of /t/, where the tongue presses the alveolar region up until the end, where the tongue tip is lowered for the production of /u/. Figure 12 shows the temporal evolution of the articulator positions in the five planes. For example, by visually comparing the tongue position between midsagittal and adjacent planes (e.g., frame 9), one can notice that the tongue is lower in the midsagittal plane near the teeth region. Additionally, for most of the images of the R1 and L1 planes, lips are almost closed, in contrast to the midsagittal plane, where they are clearly open. This information cannot be derived from the midsagittal frames alone. The results of the normalized image similarity before and after the application of the atlas are presented in Figure 11.

## 5. Discussion

**Figure 13:**
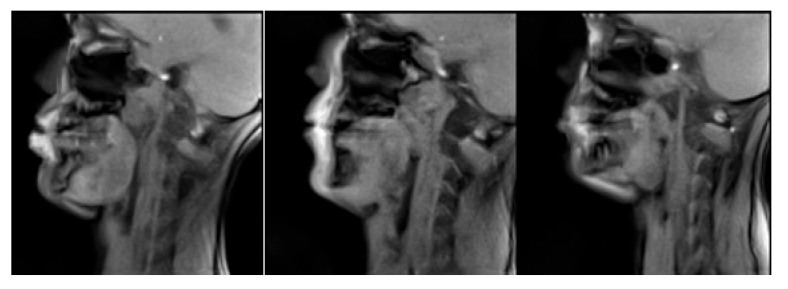
Original L2 frames during /u/ for speakers 6–8 (**left** to **right**). One can notice that images in this plane are a bit blurrier compared to the midsagittal plane (Figure 12, row 5).

Images of the R2 and L2 planes are blurrier compared to the other planes due to the fact that the original images of the speakers at that plane (Figure 13) suffer from a “partial volume effect” [38]. Indeed, the slice thickness is 8 mm, and when moving away from the midsagittal plane, the volume of one pixel may correspond to a mixture between more than one type of tissue (muscles, fat, teeth) and air, which give rise to some blurring (see Figure 12 row 5). However, one can still extract useful information about the movement of articulators such as the tongue body.

**Figure 14:**
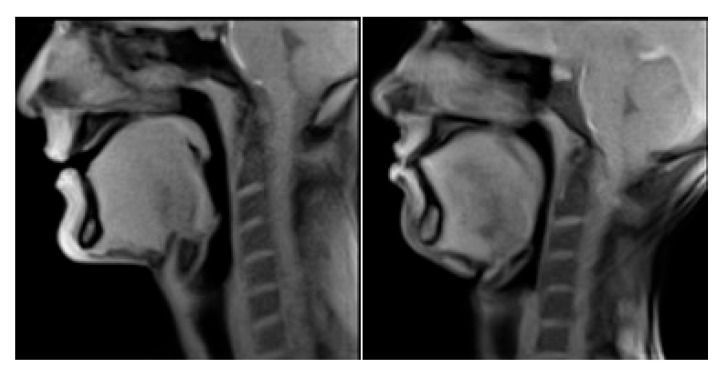
Silence frames for two speakers. One can see that more vertebras are visible for speaker 5 (**left**) compared to speaker 6 (**right**).

By comparing the atlas images against the individual subject’s images, one can notice that atlas images are less sharp. This could be due to histogram matching that took place before every image transformation or to the initial histogram matching of all the silence frames with their average histogram. It could also be due to the interpolation kernel during the spatial transform or because of the image averaging procedure both during silence creation and during the atlas sample generation. Additionally, another reason is that at step 2 of the atlas construction process (when the subject independent anatomical space is created), there is some loss of sharpness due to anatomical and head posture differences (Figure 14). Even though the reference silence image does not look strongly connected with the final atlas synthesized images, any loss of sharpness could further propagate. Indeed the silence frame was used as a reference to match the histograms and was also used to transform all the dynamic data of all subjects in order to remove subjects’ anatomical information and create “anatomically neutral” dynamic data.

The second noticeable point is that the spine is not very sharp in some cases for two reasons. This region is also affected by the general loss of sharpness, but the main reason is that posture and anatomical differences between subjects, especially between males and females, resulting in more vertebrae being visible for some subjects and less for others (see Figure 11 row 3). This probably affects the transformation algorithm since these extra vertebras have no place to be directly mapped. They are therefore compressed or extended in the opposite case, within the spine. However, we can see that the main articulators, such as the tongue, are not strongly affected. Even if there is no objective criterion that specifically focuses on the articulators since every image was treated as a whole, this behavior was expected because all the images contained the whole vocal tract, and thus, the impact of moving articulators is indirectly stronger on the transformations computed compared to that of some vertebra (C6) that sometimes appears and sometimes not. Additionally, the similarity criterion that was used for image registration [33] is mutual information which further supports the visual observations.

The first use of the atlas concerns the highlighting of average or speaker-specific articulatory strategies. The measurement of the similarity between the speaker’s images registered on the atlas and the atlas images is a way to detect these articulatory strategy deviations. The second potential use concerns the study of the dynamic 3D area function [19] since it allows the use of one representative subject, i.e., the atlas, instead of one random subject. The advantage is that one could use the method proposed by those authors directly on the atlas in order to get generic results, preventing us from having to extract area functions from several subjects and then combine them, which is the common strategy so far. 

Another use of the atlas concerns the transformation of 2D rtMRI videos into 3D dynamic videos [39,40] since the atlas incorporates the real 3D dynamic information that occurs during the production of continuous speech and not just estimates it from static 3D and midsagittal rtMRI. By using the atlas, one can directly extract the 3D shape of the vocal tract by using the stacks of the parallel sagittal images and use them to calculate transformations from the midsagittal plane to the parasagittal planes. They can be used to find estimations of the 3D dynamic shape of the vocal tract by using only the midsagittal plane. Such videos would allow the complex tongue constriction events to be investigated in depth [26].

Automatic tracking of the vocal tract contours [41,42] could also take advantage of the atlas to map a specific subject data whose data have to be delineated. The main advantage is that once the atlas is created, it could be used to process new rtMRI data without requiring data preprocessing every time, retraining models, etc. Finally, the main contribution of this work is that the atlas is a true golden speaker who embodies the speaker’s independent articulatory gestures.

## 6. Conclusions

In summary, this paper presents a method for creating a dynamic 3D atlas of the vocal tract that can be used as a reference space for studying speech production. Two-dimensional rtMRI data on parallel planes were combined using piece-wise linear alignment and the adaptive Gaussian kernel method to synthesize the images of the final atlas. The main contribution is to incorporate the speaker variability directly in the construction of the atlas. This approach almost removes inter-speaker variability of the resulting space, therefore providing a generic speaker model. Since any speaker can be “projected” onto this generic speaker, a direct extension consists in transforming one speaker into another using the atlas as a pivot with the anatomical adaptation on the one hand and the temporal adaptation, i.e., finer articulatory strategy aspects, on the other hand. This could be particularly useful to exploit resources that exist for one or a few speakers only. For instance, when 3D area functions were acquired for one speaker, the mapping between this speaker and the generic speaker gives a mapping that can then be used for any speaker by using the generic speaker as a pivot. This solution gives a more robust mapping than what could be performed for each pair of speakers independently. Another application would consist of investigating language-specific articulatory strategies by exploiting atlases built for several languages. The comparison of the language atlases would enable invariant articulatory features imposed by anatomy to be separated from language-specific strategies.

A limited number of CVs was involved in this study, and an ambitious perspective would be to incorporate all the phonetic contexts of a language, i.e., all VCVs, CVs and CCVs, in order to be able to exhaustively cover the articulation of the target language. The recording of all the contexts required for eight speakers and five planes, together with the corresponding fine temporal annotations required to build the global atlas, is unrealistic. A perspective thus would consist of defining a minimal set of sequences used to build an atlas, which would nevertheless be able to cover the articulation of the target language exhaustively and also provide efficient coarticulation modeling. 

## Figures and Tables

**Table 1:** VT Measurements.

Speaker	Length (mm)	Height (mm)	Width (mm)
SP1	97	92	40
SP2	77	76	32
SP3	99	81	40
SP4	89	69	34
SP5	94	86	36
SP6	87	81	32
SP7	88	90	38
SP8	87	67	34
Mean	89.8	80.3	35.8
SD	6.5	8.6	3.1

**Table 2:** Average phoneme duration (in number of frames at 50 fps.

Syllable	C	V	CV
fi	9	5.65	14.65
fa	8.175	6.475	14.65
fu	7.525	6.9	14.425
pi	6.55	7.275	13.825
pa	7.475	8.55	16.025
pu	6.6	7.625	14.225
si	8.775	5.875	14.65
sa	8.9	6.05	14.95
su	9.025	5.2	14.225
ti	7.6	6.825	14.425
ta	6.85	6.7	13.55
tu	7.025	4.85	11.875

**Table 3:** Adaptive Gaussian kernel method parameters.

Parameter	Value	Description
k	7	Number of samples
μ	τ	Selected time point for synthesis
σ	$\sqrt{-{\left(\tau -\tau_{f}\right)}^{2}/\left(2*ln\left(0.35\right)\right)}$	Standard deviation

**Table 4:** Cross validated results. From left to right: CV, average similarity score before the use of atlas, the standard deviation of the average similarity before the use of atlas, average similarity after the use of atlas, standard deviation of the average similarity after the use of atlas.

Phoneme	Mean (Before)	SD (Before)	Mean (After)	SD (After)
fi	0.872	0.044	0.975	0.014
fa	0.876	0.047	0.976	0.014
fu	0.869	0.043	0.974	0.015
pi	0.874	0.044	0.976	0.015
pa	0.874	0.046	0.975	0.014
pu	0.873	0.040	0.974	0.015
si	0.872	0.044	0.975	0.014
sa	0.870	0.044	0.974	0.019
su	0.873	0.045	0.976	0.016
ti	0.873	0.046	0.974	0.016
ta	0.877	0.048	0.976	0.016
tu	0.874	0.044	0.975	0.021
